# Epigenetic Remodeling of Regulatory Regions by Indicaxanthin Suggests a Shift in Cell Identity Programs in Colorectal Cancer Cells

**DOI:** 10.3390/ijms26136072

**Published:** 2025-06-24

**Authors:** Maria Antonietta Ragusa, Carla Gentile, Aldo Nicosia, Salvatore Costa, Sara Volpes, Laura Greco, Flores Naselli, Fabio Caradonna

**Affiliations:** 1 Dipartimento di Scienze e Tecnologie Biologiche Chimiche e Farmaceutiche (STEBICEF), Sezione di Biologia Cellulare, Università di Palermo, Viale delle Scienze, Ed. 16, 90128 Palermo, Italy; maria.ragusa@unipa.it (M.A.R.); carla.gentile@unipa.it (C.G.); salvatore.costa@unipa.it (S.C.); sara.volpes@unipa.it (S.V.); laura.greco08@unipa.it (L.G.); fabio.caradonna@unipa.it (F.C.); 2 Institute for Biomedical Research and Innovation-National Research Council (IRIB-CNR), 90146 Palermo, Italy; aldo.nicosia@irib.cnr.it; 3 Dipartimento di Scienze della Terra e del Mare, Università di Palermo, 90123 Palermo, Italy; 4 National Biodiversity Future Center (NBFC), 90133 Palermo, Italy

**Keywords:** Indicaxanthin, phytochemical, Methylomic signature, colorectal cancer

## Abstract

Aberrant DNA methylation is a hallmark of colorectal cancer (CRC), contributing to tumor progression through the silencing of tumor suppressor genes and activation of oncogenes. Indicaxanthin (IND), a dietary betalain pigment from *Opuntia ficus indica*, has shown antiproliferative effects in CRC models, yet its epigenetic impact remains unexplored. In this study, we investigated the effects of IND on the methylome of Caco-2 cells using Reduced Representation Bisulfite Sequencing (RRBS). IND induced a global hypermethylation profile, particularly at gene promoters and CpG islands. Among the differentially methylated genes, 60% were protein-coding, and 10% encoded transcription factors, including *PAX5* and *TFAP4*, both hypermethylated at active enhancers. Functional enrichment analysis revealed pathways beyond canonical intestinal functions, suggesting altered cell identity and plasticity. Transcription factor targets (*SOX10*, *NFKB1*, *AHR*, *ARNT*) were significantly enriched among the affected genes, several of which are involved in transdifferentiation processes. Methylation changes also indicated potential reprogramming toward epithelial cell types from pulmonary or neuroectodermal origin. Moreover, IND induced selective hypomethylation of Alu elements on chromosome 21 and hypermethylation of rDNA loci, hinting at suppressed ribosomal biogenesis. Overall, these findings highlight the epigenetic remodeling potential of IND and its possible role in modulating cell fate and metabolism in CRC cells.

## 1. Introduction

Colorectal cancer (CRC) poses a significant global health threat, ranking as the third most commonly diagnosed cancer worldwide, with an estimated 1.9 million new cases diagnosed in 2020 alone [1]. This translates to a substantial burden on healthcare systems and a devastating impact on individuals and families. Aberrant DNA methylation, a key epigenetic modification, plays a critical role in CRC development. DNA methylation involves the addition of a methyl group to specific DNA sequences. This modification alters DNA structure and consequently the binding affinity of transcription factors, architectural proteins, chromatin remodelers and readers and therefore can change DNA functionality [2,3]. In the context of CRC, abnormal methylation patterns can result in the silencing of tumor suppressor genes, which normally act as brakes on cell growth or in the activation of oncogenes, genes that promote uncontrolled cell proliferation, further contributing to tumor formation and progression [4]. Therefore, studying changes in DNA methylation in CRC is crucial for developing novel diagnostic and therapeutic strategies.

The human epithelial cell line, Caco-2, is a well-established and widely used in vitro model system that mimics the intestinal epithelium [5]. Derived from a human colorectal adenocarcinoma, Caco-2 cells possess the remarkable ability to spontaneously differentiate into mature enterocyte-like cells after reaching confluence in culture, typically within two weeks [5]. These differentiated Caco-2 cells exhibit a remarkably normal intestinal epithelial phenotype and have proven to be a valuable tool for researchers studying intestinal uptake, nutrient and drug transport, and various cellular processes relevant to small intestine function and pathophysiology [6,7].

Betalains, a recently discovered class of plant chemicals, are found only in nine out of twelve families within the Cariophyllales order. Beetroot (Beta vulgaris) and fruits of prickly pear cacti (Opuntia species), particularly Opuntia ficus indica, are the primary sources of these pigments [8]. Research exploring the antitumor potential of betalains has utilized both in vitro and in vivo models [6]. Indicaxanthin (IND), a yellow pigment extracted from *Opuntia ficus indica*, demonstrates high bioavailability in humans when consumed in its natural form [9]. Scientific evidence highlights its free radical scavenging and antioxidant properties [10]. Additionally, studies have shown IND’s anti-inflammatory effects on intestinal epithelial cells, both in vitro and in mouse models of inflammation [11]. Notably, our research has demonstrated the cytotoxic activity of IND against various colorectal cancer cell lines, including Caco-2 cells [12,13,14].

Despite its documented bioactivity, the rationale for exploring Indicaxanthin through an epigenetic lens stems from emerging evidence suggesting that certain dietary compounds can influence gene expression not only via canonical signaling pathways, but also through the modulation of the epigenetic landscape [15,16]. Given IND’s proven antioxidant and anti-inflammatory effects, we hypothesized that it might also contribute to altering DNA methylation patterns, potentially affecting gene regulatory mechanisms in CRC.

Epigenetic modulation by dietary compounds represents a key mechanism in the field of nutrigenomics, which investigates the interplay between nutrition and gene expression. Nutrigenomics aims to understand how specific bioactive food components can modulate epigenetic marks—such as DNA methylation and histone modifications—thereby influencing cellular phenotypes and disease susceptibility [17,18]. In this context, exploring Indicaxanthin’s potential as an epigenetic modulator may provide novel insights into its mechanism of action and relevance in CRC prevention or therapy.

This study aimed to investigate the potential of Indicaxanthin to modulate the DNA methylation landscape in Caco-2 colorectal carcinoma cells. To comprehensively understand Indicaxanthin’s influence on DNA methylation, we employed the Reduced Representation Bisulfite Sequencing (RRBS) method. The data were used to identify differentially methylated regions (DMRs) upon Indicaxanthin treatment compared to controls. A bioinformatic analysis of the DMRs was conducted to elucidate their functional roles in colorectal cancer development, revealing key pathways sensitive to Indicaxanthin’s action. Our findings on the modulation of methylation patterns across the genome offer intriguing insights into the potential molecular mechanisms by which Indicaxanthin exerts its recently observed antiproliferative and pro-autophagic effects on various tumor cell lines.

## 2. Results and Discussion

### 2.1. Indicaxanthin-Induced Methylation Changes in Caco-2 Cells

Caco-2 cells were treated with three different concentrations of IND, as described in the Materials and Methods section.

**Figure 1:**
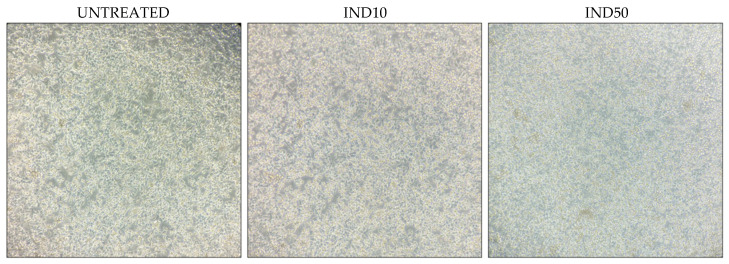
Representative images of Caco-2 cells under different treatment conditions (untreated or treated with IND10-IND50). Images were captured after 48 h of treatment. No morphological changes were observed in response to increasing concentrations of IND.

First of all, we aimed to assess whether the different treatments with IND induced any morphological changes in Caco-2 cells. In Figure 1, Caco-2 cells in culture are shown, both untreated and treated with IND 10 and IND 50. As observed from the images, there are no significant morphological differences between the untreated control cells and those treated with IND10 or IND50.

**Figure 2:**
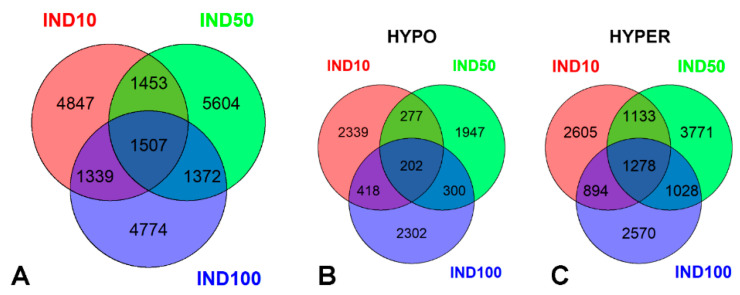
Venn diagrams showing the correspondence between the three IND treatments: (**A**) all DMRs, (**B**) DMR hypo, and (**C**) DMR hyper.

RRBS data from the control (untreated cells) and treated samples were analyzed in a pairwise fashion to determine differentially methylated CpGs and tiles. All CpGs and tiles were assembled in differentially methylated regions (DMRs) 200 bp long, and DMCs were filtered so that only those with at least four DMCs in a DMR were kept. In total, 9146, 9936 and 8992 differentially methylated regions (DMRs) were identified in Caco-2 cells treated with IND10, IND50 and IND100, respectively (|df| ≥  25% and q value < 0.01). Venn diagrams in Figure 2 show DMR numbers for regions that gain or lose DNA methylation after IND treatments (hyper and hypo) and correspondence between the three treatments.

Although the total number of DMRs is comparable across the three datasets, hypomethylated tiles represent a lower percentage, particularly in IND50 (IND10: 35%, IND50: 27%, IND100: 36%). Additionally, the percentage of hypermethylated DMRs shared among all three treatments is much higher than that of hypomethylated DMRs (86% hypermethylated vs. 14% hypomethylated). These findings highlight and confirm the hypermethylating effect of IND, particularly at 50 µM [14].

### 2.2. Epigenetic Impact of Indicaxanthin on Promoter and Regulatory Regions

In order to gain functional insights from methylation data, all DMRs were annotated with respect to genomic features. In particular, genes (distance to TSS, promoter, exons, introns), CpG islands (CGI), cCRE, and TSS CAGE general features were annotated, together with features specific for Caco-2 cell lines (DNAseI hypersensitivity, CTCF binding sites, and histone PTMs (H3K4me1, H3K4me3, H3K9me3, H3K36me3).

Since a 48 h treatment of proliferating Caco-2 cells with IND induced a concentration-dependent inhibition of cell growth with an IC_50_ of 115 µM [13], previous results led us to consider that treatment with IND100 could be excessively stressful for the cells, potentially impairing their ability to adequately respond to the phytochemical [14]. Moreover, IND50 treatment causes the strongest hyper methylating effect. Based on these observations, we analyzed the RRBS data by focusing on the DMRs shared between the IND10 and IND50 treatments.

Based on the transcription start site (TSS) annotation generated by the FANTOM project, 1.3% of FANTOM5-mapped TSS were found differentially methylated. Additionally, 6% of promoter regions (from −1000 to +1000 with respect to TSS) overlap with at least one DMR.

Considering the common DMRs shared between IND10 and IND50 treatments, and selecting those annotated as promoters or cCREs, analysis of the annotated DMRs revealed, as expected, a high proportion of differentially methylated regions located in promoters and candidate cis-regulatory elements (CCREs) characterized by histone tail modifications associated with promoter and enhancer activity in Caco-2 cells (H3K4me1 and me3). Conversely, only a small fraction of DMRs mapped to regions marked as transcribed or repressed, both among hypermethylated and hypomethylated sites relative to controls. Notably, hypermethylated DMRs exhibited slightly higher enrichment at regions with high DNase I hypersensitivity compared to hypomethylated DMRs, suggesting potential remodeling of chromatin accessibility in regulatory elements (Table 1).

Out of the 3253 DMRs annotated as promoters or cCREs, a total of 2565 unique genes were found to be differentially methylated, the majority of which (62%) are protein coding genes (Table 2).

**Figure 3:**
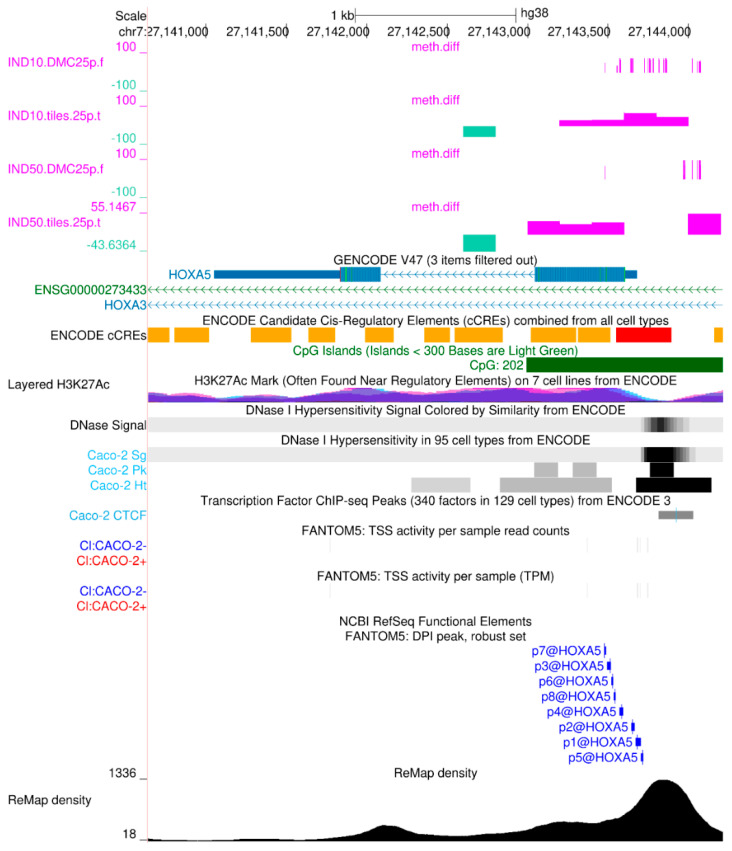
Effect of Indicaxanthin on HOXA5 methylation. The HOXA5 gene shows promoter hypermethylation and a hypomethylated candidate cis-regulatory element (cCRE) following Indicaxanthin treatment. The top tracks display raw methylation differences derived from the analysis of differentially methylated cytosines (DMCs) and 200 bp tiles. Several tracks shown are default features of the UCSC Genome Browser, while others have been specifically activated to highlight functional genomic data from Caco-2 cells.

A representative genome browser snapshot (UCSC) illustrating RRBS-derived methylation data alongside publicly available datasets used for functional genomic annotation is shown in Figure 3. This visualization highlights the genomic context of differentially methylated regions (DMRs), including their overlap with promoter elements, enhancers, DNase I hypersensitivity, and histone modification marks, supporting their potential regulatory relevance.

**Figure 4:**
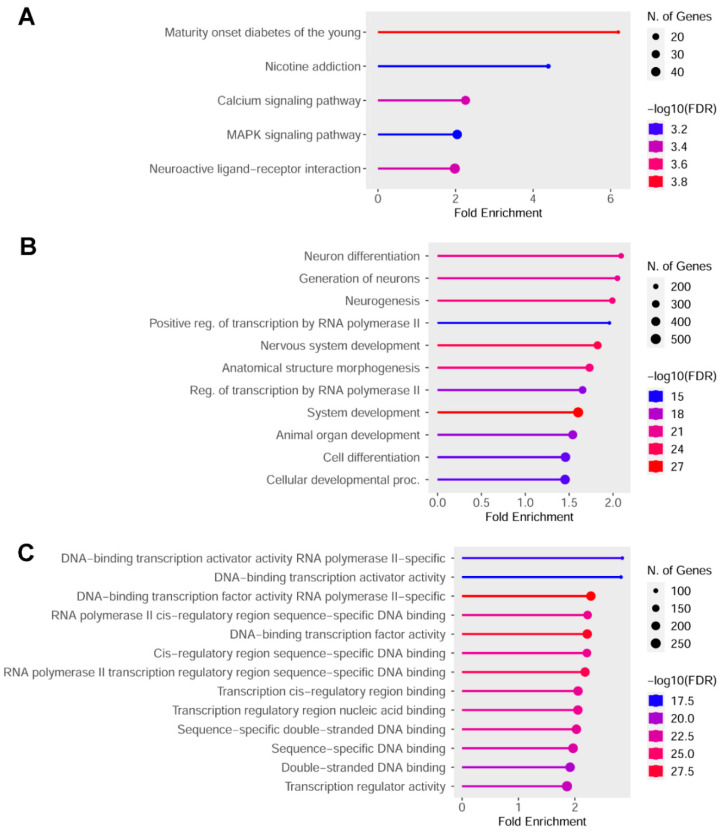
Gene-set enrichment analysis performed on the gene list differentially methylated in promoter or cCRE. (**A**) KEGG enrichment analysis; (**B**) GO biological process enrichment analysis; (**C**) GO molecular function enrichment analysis.

KEGG pathway enrichment analysis (FDR cutoff = 0.005) revealed significant associations with various signal transduction pathways as well as with pathways unexpectedly related to cell types other than intestinal cells. Gene Ontology enrichment analysis (FDR cutoff = 0.5 × 10^−14^) highlighted biological process terms predominantly associated with cell differentiation, development, and transcriptional regulation. Regarding molecular function, the most significant terms were related to transcription factor activity. Consistently, the most enriched term under the cellular component category was chromatin (*n* = 195, FDR = 8.0 × 10^−20^, Fold enrichment = 2.1), suggesting a strong epigenetic impact of the treatment on regulatory regions involved in gene expression control (Figure 4).

### 2.3. Top Hypomethylated and Hypermethylated Targets Induced by Indicaxanthin

To further investigate the biological significance of the DNA methylation changes induced by Indicaxanthin, DMRs were filtered based on their annotation as promoters and separated in two groups: less methylated (“hypo”) and more methylated (“hyper”) with respect to untreated controls. These were ranked by differential methylation (diff. meth) and two tables were generated, reporting the top genes exhibiting the strongest promoter hypomethylation and hypermethylation in both IND10 and IND50 treatments (Table 3 and Table 4).

Among the top 12 most demethylated genes following Indicaxanthin treatment, *SHH* stands out as a central regulator of the Hedgehog (Hh) signaling pathway [19,20]. Notably, other key components of this pathway, including *GLI3* and *BCL2*, were also found as demethylated, alongside downstream targets such as *NKX2-2* and *DBX2*. In contrast, *PAX6*, a known SHH-repressed gene, exhibited increased promoter methylation. This epigenetic pattern mirrors the developmental logic of Hh signaling, wherein SHH activation represses class I genes (*PAX6*, *DBX2*) and promotes class II effectors (*NKX2-2*), along with anti-apoptotic genes like *BCL2*. Although Hh signaling is generally inactive in colorectal cancer cell lines, including Caco-2 [21], these findings suggest that Indicaxanthin may trigger a partial reactivation of a developmental-like transcriptional network via targeted DNA demethylation.

Other highly demethylated genes included *ONECUT1* (a regulator of endodermal differentiation) [22], *PROK2* (implicated in neuronal guidance and inflammation) and *DIO3* (a thyroid hormone-inactivating enzyme linked to developmental timing). Together, these data indicate a broader epigenetic reprogramming of genes associated with differentiation, morphogenesis and neuro-immune crosstalk. These observations support the hypothesis that Indicaxanthin modulates the epigenome in a manner that may influence cell identity and plasticity beyond canonical intestinal functions.

Efforts were made also for accounting genes showing hypermethylated promoters following Indicaxanthin treatment. Among them, several are noteworthy for their involvement in cell signaling, differentiation, and tumor-related processes. For instance, STAT5A, a transcription factor involved in cytokine signaling and immune regulation [23], and TIMP2, a well-known inhibitor of metalloproteinases implicated in extracellular matrix remodeling and cancer progression [24], suggest potential repression of proliferative and invasive pathways. Similarly, hypermethylation of *MSX2* and *BARX2*, both transcription factors associated with developmental programs and epithelial differentiation, may reflect the epigenetic silencing of lineage-specific transcriptional networks [25,26].

Furthermore, the hypermethylation of *NAPRT* (a key enzyme in NAD biosynthesis), *PTGIS* (involved in prostaglandin metabolism) and *UGDH* (involved in glycosaminoglycan biosynthesis) points to a putative modulation through the downregulation of cellular metabolism and inflammatory signaling. Taken together, these epigenetic changes suggest that Indicaxanthin may repress pathways supporting proliferation, immune evasion, or metabolic adaptation, possibly contributing to a shift toward a quiescent or differentiated cellular state.

### 2.4. Indicaxanthin Targeted the Epigenetic Landscape of Several Transcription Factors

Since among the genes differentially methylated following treatment with 10 µM and 50 µM Indicaxanthin, nearly 10% correspond to transcription factors, we performed a focused analysis on this subset.

**Figure 5:**
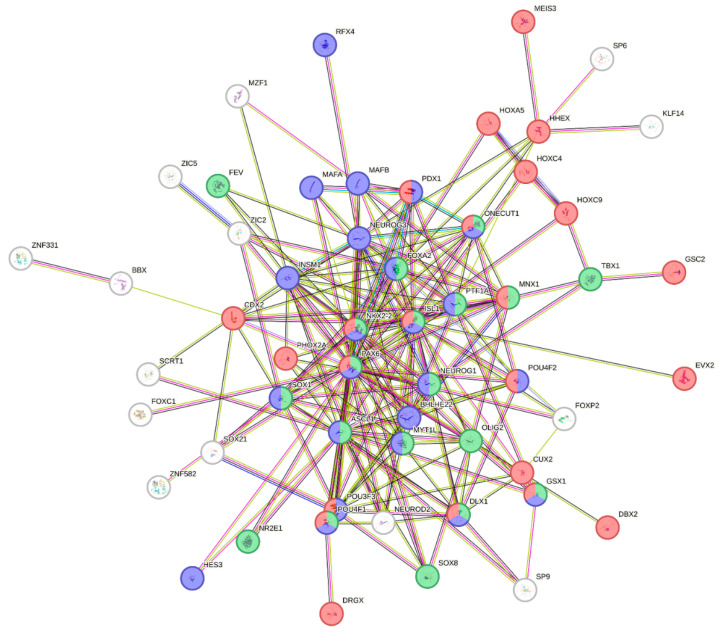
Representative cluster #1 (Cluster “Mixed, incl. Neuron fate commitment, and Maturity onset diabetes of the young”—Pancreas development: *n* = 55/225. PPI enrichment *p*-value: < 1.0 × 10^−16^) of the results of k-means clustering analysis performed with STRING. Functional enrichments are colored: green: Cell fate commitment; blue: “Neuron fate commitment, and Regulation of beta-cell development” STRING cluster; red: Homeobox proteins.

Analysis of these transcription factors revealed that some are involved in transdifferentiation processes. Notably, pioneer factors, homeobox proteins, and regulators of cell differentiation (Cluster “Cell type specification”: *n* = 43) were identified; in particular enrichment analysis (see STRING results) revealed association with pancreatic, muscle, and neuronal cell lineages/differentiation programs (Cluster “Mixed, incl. Neuron fate commitment, and Maturity onset diabetes of the young”—Pancreas development: *n* = 55; Figure 5).

**Figure 6:**
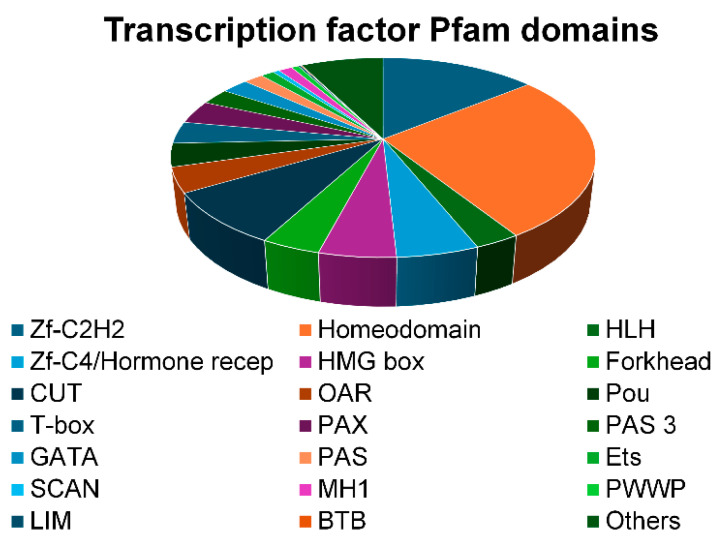
Pie chart showing TF classes, proportionally to the enrichment for each class.

Analysis of PFAM domains among the most represented transcription factor domains/classes, also in proportion to their enrichment relative to the total number of genes within each class, revealed a predominance of Zf-C2H2 and Homeodomain classes, followed by Zf-C4/Hormone Receptors, Forkhead, and HMG Box domain. Notably, four out of seven members of the *CUT* family are epigenetically modified: *SATB2* and *ONECUT1* were hypomethylated, while *CUX2* and *CUX1* were hypermethylated, suggesting selective remodeling of transcriptional networks involved in differentiation programs (Figure 6).

To further elucidate the regulatory landscape affected by Indicaxanthin treatment, we investigated the enrichment of transcription factor targets within the list of differentially methylated genes. This analysis revealed a notable enrichment of genes regulated by several key transcription factors, suggesting that Indicaxanthin may influence broader transcriptional programs through epigenetic remodeling. Specifically, enrichment analysis of transcription factor targets within the differentially methylated gene list revealed significant over-representation of *ARNT* and *AHR* target genes, along with enrichment for *PAX5, SOX10* and *TFAP4* target genes (Table 5).

AHR (aromatic hydrocarbon receptor) is a ligand-activated transcription factor that, upon binding environmental, dietary, microbial, or metabolic ligands, translocates to the nucleus and forms a heterodimer with ARNT (Aryl Hydrocarbon Receptor Nuclear Translocator) to activate gene transcription via xenobiotic response elements (XRE). This AHR-ARNT complex regulates processes such as angiogenesis, hematopoiesis, metabolism, immune modulation, and cancer progression [27,28]. AHR also represses the circadian gene *PER1* by inhibiting CLOCK-BMAL1 activity [29].

ARNT not only partners with AHR but also forms a heterodimer with EPAS1 (HIF2A), binding hypoxia response elements (HRE) to regulate genes involved in hypoxia adaptation, vascular development (e.g., *VEGF* expression), and blood–brain barrier formation [30]. Additionally, AIP (AH receptor-interacting protein), whose gene is differentially methylated, may enhance AHR signaling by modulating ligand sensitivity or nuclear translocation.

Moreover, the *AHRR* (aryl hydrocarbon receptor repressor) gene is also differentially methylated in some regulatory regions and the protein encoded by this gene functions as a feedback modulator by repressing AhR-dependent gene expression [31].

Moreover, differentially methylated regions (DMRs) resulting from Indicaxanthin treatment are significantly enriched in genes targeted by key transcription factors such as PAX5, SOX10, TFAP4 and NFKB1. Notably, both *PAX5* and *TFAP4* themselves are hypermethylated at active enhancer regions marked by H3K4me3, suggesting a potential epigenetic silencing of upstream regulatory hubs. These factors are known to orchestrate crucial processes such as lineage specification (PAX5, SOX10), regulation of cell proliferation and differentiation (TFAP4, a regulator of epithelial–mesenchymal transition) and inflammatory signaling (NF-κB). In line with the observed enrichment in *NFKB1* targets among differentially methylated genes, previous studies have shown that betanin, a betalain structurally related to Indicaxanthin, reduces NF-κB expression in the brain and promotes phosphorylation of the inhibitory protein IκBα, thereby dampening NF-κB activation [32]. Furthermore, Indicaxanthin itself has been reported to bind and inhibit the active form of the IκB kinase (IKK) complex, leading to NF-κB sequestration and attenuation of its pro-inflammatory effects [33]. These findings support the hypothesis that the epigenetic impact of Indicaxanthin may extend to the modulation of key inflammatory pathways through direct or indirect inhibition of NF-κB signaling.

PAX5, a member of the Paired box protein (PAX) family, plays a crucial role in the early specification of cell fate and the subsequent morphogenesis of various tissues and organs [34]. Notably, five out of nine *PAX* family members (*PAX1, PAX2, PAX3, PAX5* and *PAX6*) are differentially methylated following IND treatment. PAX3 is known to act synergistically with SOX10, which is expressed in fetal brain and in adult brain, heart, small intestine and colon [35,36,37,38]. Interestingly, *UNCX* (Homeobox protein unc-4 homolog), a gene encoding a transcription factor involved in somitogenesis and neurogenesis, is differentially methylated and may act upstream of *PAX9,* suggesting a broader developmental epigenetic response.

TFAP4 has been reported to be a regulator of EMT, which is a foundation for the metastasis of colorectal cancer cells. The expression of *TFAP4* mRNA is upregulated in CRC tissues compared to tumor-adjacent tissues, and EMT and metastasis of Caco-2 cells are enhanced by TFAP4 [39].

The coordinated targeting of these transcription factors could imply that Indicaxanthin may exert broad regulatory control over transcriptional networks governing cell identity and immune responsiveness through selective DNA methylation changes. This is consistent with the observed enrichment of DMRs in pathways related to transcriptional regulation and cell fate determination.

### 2.5. Analysis of Cell Type Signature (Transcription Factor TSS Expression)

Cell phenotypes arise from sequential gene expression programs that govern specification and differentiation. Within these genetic programs, specific transcription factors (TFs) are either activated or repressed, orchestrating regulatory signals at both local and distal levels. These signals converge at transcription start sites (TSSs), where they control gene activation by modulating promoter activity. Consequently, a clear link exists between TF expression profiles and cell identity.

Thus, Caco-2 TFs cell signature was analyzed in response to IND exposure to test the hypothesis that IND treatment may have induced transdifferentiation programs enabling Caco-2 cells to shed their original phenotypes and adopt new identities through different usage of regulatory programs.

We recovered transcription factor TSSs with enriched expression in Caco-2 cells and compared this dataset with the CAGE TSS list of transcription factors that exhibit differential methylation following IND treatment.

Defining the promoter region as spanning −1000 to +1000 bp relative to the TSS, we identified at least 30 promoters (corresponding to 18 TFs) that are both enriched in Caco-2 cells and show increased methylation after IND treatment. Additionally, five promoters (associated with five TFs) are enriched in Caco-2 cells but display reduced methylation following IND treatment (Table 6).

**Figure 7:**
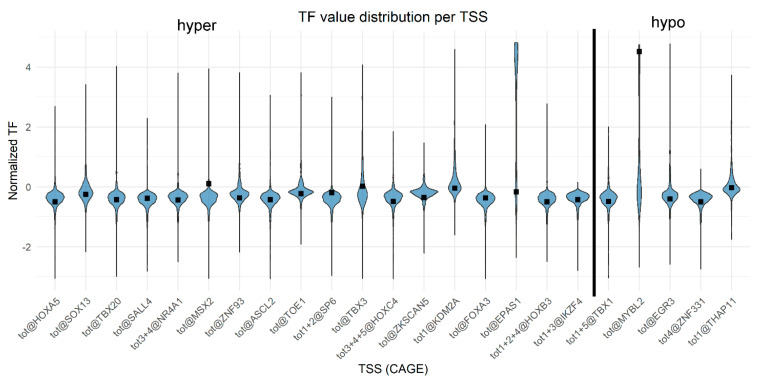
Violin plot of relative normalized TF for all samples analyzed in the Fantom 5 site (https://fantom.gsc.riken.jp/5/sstar/Browse_samples, accessed on 14 January 2025). Black squares indicate values for Caco-2 cells.

The normalized data of TSS expression (TF: Tags per Feature) from all samples (incl. primary cells, cell lines, and developmental—*n* = 889) that were profiled in the FANTOM5 project are represented in Figure 7.

Three distinct cell types were simulated, each representing a scenario with minimal, moderate, or maximal variation in transcription levels, starting from differentially methylated transcription start sites (TSSs). This approach resulted in the generation of a comprehensive list of TF utilization within 592 distinct cell types, to which the three simulated types were subsequently added. A clustering analysis was then performed on this dataset, based on the co-expression patterns of transcription factors, allowing for the identification of regulatory relationships and potential phenotypic transitions among the analyzed cell types.

As expected, Caco-2 cells cluster relatively close to prostate and intestinal epithelial cells (polarized). The simulated cells with a low effect on Caco-2 remain closely aligned with the original Caco-2 cells and are positioned within the same branch as other cancerous cell types, including ductal cells, plasma cells from chronic lymphocytic leukemia, as well as neuroblastoma and retinoblastoma cell lines. In contrast, cells simulated with a moderate or high effect on Caco-2 are located near adrenal cortex adenocarcinoma cell lines, pulmonary/bronchial epithelial cells (which originate from the endodermal lineage and contribute to the epithelial structures of the trachea, airway, and alveoli), Hep-2 cells, and the leiomyoblastoma cell line G-402 (an epithelial-like cell derived from intermediate mesoderm).

Therefore, it is reasonable to assess that changes in genomic methylation landscapes, including promoter region of the TF-related cell phenotypes, exerted by IND exposure lead Caco-2 cells to attempt the activation of lineage reprogramming pathways which may in turn enable Caco-2 cells to abandon their initial characteristics and acquire new traits by altering the utilization of regulatory mechanisms.

### 2.6. Indicaxanthin Influences Methylation Dynamics Within Repetitive Genomic Regions

**Figure 8:**
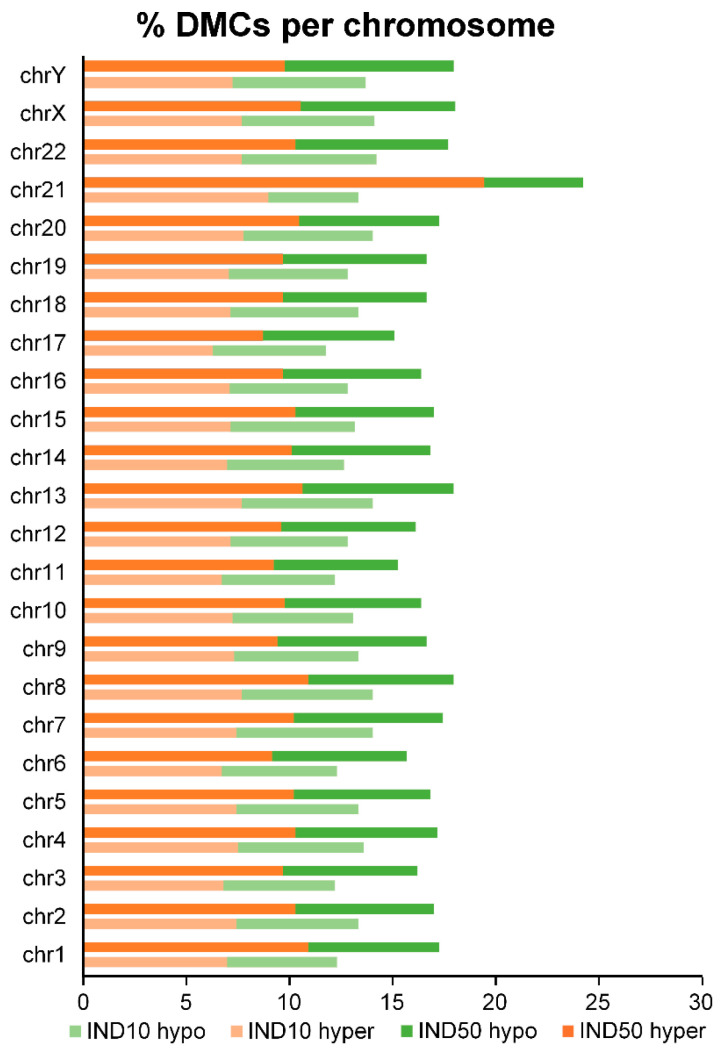
Percentage of hyper- and hypomethylated cytosines per chromosome (q value < 0.01 and methylation difference ≥ 5).

To gain further insight into the genomic architecture affected by Indicaxanthin, we examined the chromosomal distribution of differentially methylated CpGs (DMCs). Analysis of DMC distribution across chromosomes revealed a non-uniform pattern, especially following treatment with 50 µM Indicaxanthin. Notably, hypermethylated DMCs were particularly enriched on chromosome 21 (Figure 8).

Given the low gene density of chromosome 21, we investigated the potential involvement of specific repetitive sequences in driving this enrichment. Therefore, DMCs were annotated based on their localization within repetitive sequences (Table 7).

Specifically, we focused on DMCs falling within the most relevant families of repetitive elements, including Alu and MIR (SINEs), LINE-1 and LINE-2 (LINEs), microsatellites, and simple repeats.

**Figure 9:**
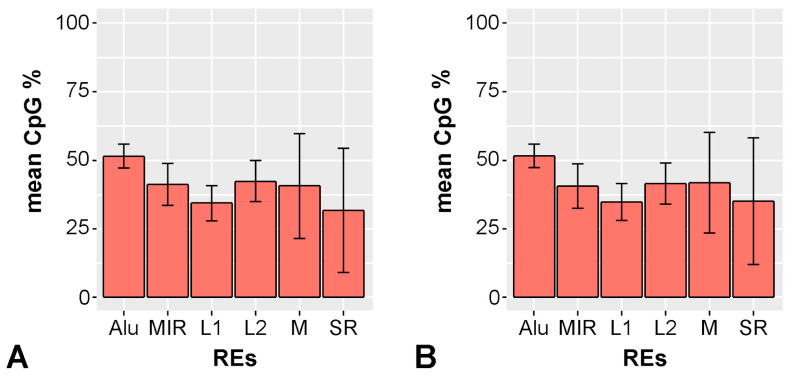
Mean CpG percentage in repetitive elements. (**A**): All genomes; (**B**): Chr21. SR: simple repeats; INR: interrupted Rpts; M: microsatellite; L1: LINE-1, L2: LINE-2; Alu: Alu element; MIR: mammalian-wide interspersed repeat.

To ensure a reliable comparison between genome-wide data with those obtained for chromosome 21, we first verified that the relative proportions of different classes of repetitive sequences in the genome were similar to those in chromosome 21 (Table 8). Furthermore, we ensured that the average CpG content within the sequences of various repeat classes was comparable between genome-wide repeats and those specifically located on chromosome 21 (Figure 9).

The data indicate that there are no substantial differences in the distribution of repetitive sequences between the whole genome and chromosome 21. This finding allows for a reliable comparison between genome-wide data and those specifically derived from chromosome 21.

**Figure 10:**
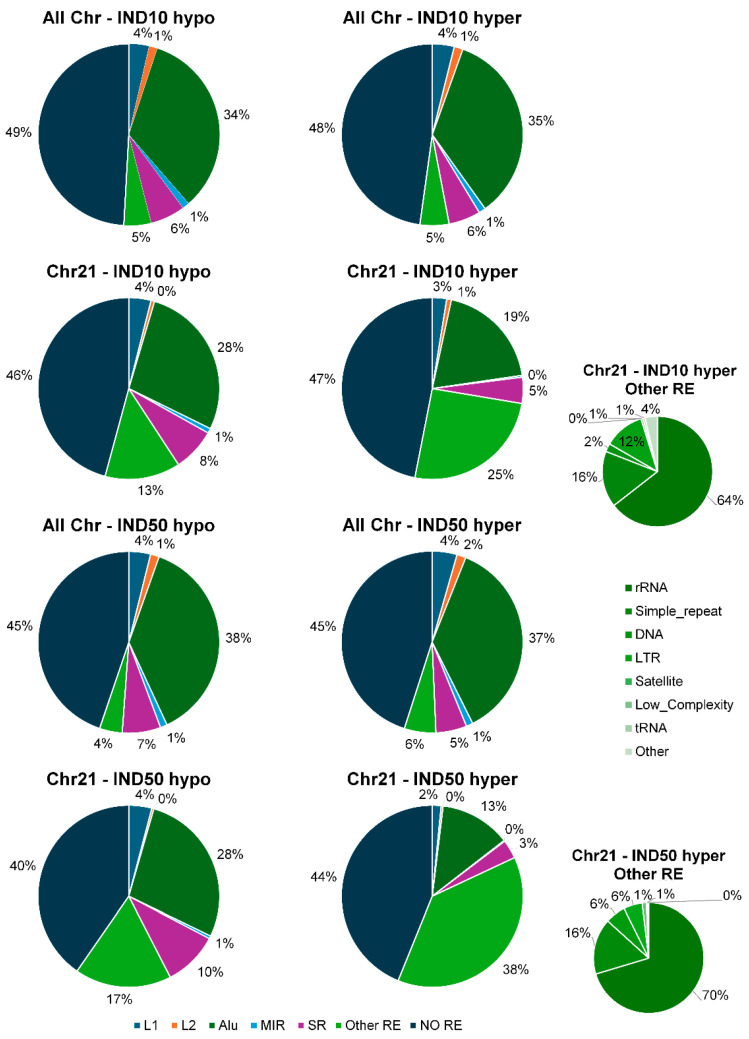
Distribution of DMCs in repetitive elements, both for genome and for chr21. The little pie charts specify the class of repetitive element indicated as “Other RE” in the big pie charts.

Subsequently, we analyzed the distribution of DMCs within the major classes of repetitive elements, both at the level of the entire methylome and specifically within the methylome of chromosome 21. As shown in Figure 10 (left panel), the proportions of hypomethylated cytosines within repetitive elements are approximately similar when comparing the whole genome to chromosome 21, for both IND10 and IND50 treatments. In contrast, hypermethylated cytosines on chromosome 21 are significantly underrepresented within SINEs (in particular Alu elements) and LINEs. Notably, Alu elements show a higher proportion of hypomethylation compared to hypermethylation, while the proportion of hypermethylated cytosines mapping to other repetitive sequences markedly increases. This effect is particularly pronounced following treatment with 50 µM Indicaxanthin, where the percentage rises from 6% to 38% (Figure 10, right panel).

These data suggest that Alu elements in chromosome 21 are more prone to demethylation in response to the same treatment, highlighting the locus-specific nature of Alu methylation dynamics. Alu elements, members of the primate-specific SINE (short interspersed nuclear elements) family, are highly abundant in the human genome. They harbor approximately 25% of all human CpG sites, the majority of which are normally methylated. However, a fraction of these CpGs remains unmethylated, and this unmethylated proportion tends to increase in cancer, indicating a loss of methylation control in pathological states. In fact, Alu hypomethylation has been shown to drive genomic instability by facilitating the accumulation of DNA damage, a mechanism linked to both tumorigenesis and epigenetic dysregulation [40,41].

Alu repeats are increasingly recognized as active participants in gene regulation and sources of transcriptional plasticity [42,43]. It has been shown that the presence of variably methylated Alu elements near promoter regions may indicate boundary plasticity, which could influence chromatin accessibility and transcription factor binding [44,45,46]. In addition to shaping the epigenomic landscape through effects on nucleosome positioning [47,48] and genome compartmentalization [49,50], Alu repeats are increasingly recognized as active participants in gene regulation and sources of transcriptional plasticity [42,43].

To further dissect the hypermethylated DMCs enrichment on chromosome 21, we examined the involvement of additional classes of repetitive sequences included in the RepeatMasker annotation dataset—namely, DNA elements, simple repeats, LTRs, satellites, low-complexity regions, tRNAs, and rRNAs. Strikingly, a disproportionately high percentage of hypermethylated cytosines was found within ribosomal RNA (rRNA) regions. This was particularly evident at 45S rRNA gene loci, prompting us to investigate the methylation status of the 5S rRNA genes located on chromosome 1. This analysis revealed that these loci are likewise hypermethylated, with an average increase in DNA methylation of approximately 28%. The rRNA genes are essential for ribosome biogenesis, and their transcriptional activity is tightly regulated by epigenetic mechanisms, including DNA methylation. Hypermethylation at these loci is known to repress rRNA transcription, thereby reducing ribosome synthesis and impairing global protein translation [51,52]. This epigenetic silencing aligns with our phenotypic findings of reduced cellular proliferation, as ribosome biogenesis is a critical driver of cell growth and division [53].

Moreover, recent evidence suggests a link between nucleolar stress—triggered by impaired rRNA synthesis—and the activation of autophagy as a compensatory survival mechanism [54,55,56]. Thus, the increased autophagic activity observed in Indicaxanthin-treated cells may reflect a cellular response to reduced ribosome availability and altered proteostasis [14]. These findings collectively suggest that Indicaxanthin may exert antiproliferative effects, at least in part, through epigenetic repression of rRNA genes, pointing to a previously unrecognized role of this dietary compound in modulating nucleolar function and cellular metabolism.

## 3. Materials and Methods

### 3.1. Cell Culture and Treatments

The Caco-2 colon adenocarcinoma cell line was cultured in high-glucose–DMEM medium plus 10% fetal calf serum (ThermoFisher, Waltham, MA, USA), 100 U/mL penicillin, 100 µg/mL streptomycin, and 2.5 mg/L amphotericin B (Invitrogen, Carlsbad, CA, USA) at 37 °C under a 5% CO_2_ atmosphere, as previously described [11,14].

IND was isolated from *Opuntia ficus indica* fruit extracts. Briefly, prickly pear fruits, collected in September-November 2001 in Sicily (Italy), were obtained from a local market and were processed within 48–72 h of collection. Four different lots of fruits, at comparable ripening stages, were analyzed for each cultivar. The fruits were peeled and finely chopped. The pulp was separated from the seeds and weighed, and 100 g pulp samples were homogenized with 100 mL of methanol. The mixtures were allowed to stand for 60 min at 4 °C before centrifugation (10 min at 3000× *g*). The organic layer was then recovered and the extraction repeated with the same volume of methanol. The combined extracts were subjected to rotary evaporation to remove the organic solvents. All the samples were portioned and stored at −80 °C [57]. Caco-2 cells were exposed to different concentrations of IND, i.e., 10 (IND10), 50 (IND50), and 100 µM (IND100), for 48 h as described in [14].

### 3.2. Reduced Representation Bisulfite Sequencing (RRBS) and Differential Methylation Analysis

RRBS is a method for unbiased DNA methylation profiling in a highly accurate manner in a genome-wide scale [58]. In this study, RRBSs were performed as described in [14].

Differential analysis was performed using *methylKit* R package (version 1.24.0), using default parameters: q-value < 0.01 and minimum coverage equal to 10. To better highlight the differences in methylation between the treated and untreated samples, differential analysis was performed on both individual CpGs and “tiles” (200 bp regions). CpG and tiles with |diff.meth| greater than 25% were considered and assembled in differentially methylated regions (DMRs) 200 bp long. Categorical flags (C, M, T, N) were introduced to identify the origin of each region: “C” denotes regions derived from clustering of DMCs, “M” refers to regions generated by *methylKit* tiling, “T” indicates regions present in both groups, and “N” refers to regions with no match in either group. Regions derived from clustering of DMCs were filtered so that only those with at least four DMCs in DMR were kept.

To facilitate the comparison of DMRs derived from the three treatments, a set of logical flags (TRUE/FALSE) was generated to indicate whether differentially methylated regions (DMRs) were shared across treatments.

Using the R package *genomation* (version 1.30.0), filtered DMRs were annotated based on genes, CGI and CGI shores (defined as regions 2 kb in length adjacent to CGIs), cCRE [ENCyclopedia Of DNA Elements (ENCODE)]. We defined promoters as the region 1 kb upstream to 1 kb downstream of gene transcription start sites (TSSs). Moreover, DNA accessibility (DNaseI hypersensitivity sites: DHS) and CTCF binding sites and histone post-translational modification in Caco-2 cells data were also used for the annotation. The data were downloaded from UCSC Hg38 genome browser tracks or from ENCODE portal (https://www.encodeproject.org/ accessed on 14 January 2025). Unless otherwise specified, all database resources were accessed between January 2024 and March 2025. Track names and dataset identifiers are included in Table 9.

### 3.3. Functional Analysis of Differentially Methylated Regions

KEGG pathway enrichment and Gene Ontology (GO) analyses were performed using the ShinyGO (v0.82 http://bioinformatics.sdstate.edu/go/, accessed on 14 January 2025) tool to investigate the functional relevance of differentially methylated regions (DMRs) [59]. Genes associated with DMRs were submitted to ShinyGO, and enrichment was assessed for biological processes, molecular functions, cellular components, and canonical pathways. A false discovery rate (FDR) threshold was applied to identify significantly enriched terms and pathways, as indicated in the Results and Discussion section.

### 3.4. Statistical Analysis

All statistical analyses were performed using R programming language (version 4.2.2). Statistical difference in methylation rates of CpG sites across treatments was determined using the Wilcoxon signed-rank test and were implemented using methylKit package (version 1.24.0).

### 3.5. Transcription Factor Analysis

K-means clustering analysis on the 225 differentially methylated genes belonging to “DNA-binding transcription factor activity RNA polymerase II-specific” term (GO:0000981) were analyzed with STRING (version 12.0—https://string-db.org).

Transcription factor domains/classes study was performed by enrichment analysis using Pfam Domains 2019 database using Enrichr platform (https://maayanlab.cloud/Enrichr/enrich (accessed on 15 January 2025)).

The enrichment analysis in transcriptional regulatory networks was performed using the ShinyGO platform using the Pathway TF.Target.RegNetwork database (data accessed from ShinyGO v0.82 in January 2025).

### 3.6. Analysis of Cell Type Signature

FANTOM5 SSTAR (Semantic catalog of Samples, Transcription initiation And Regulators) provide a way to explore samples, transcriptional initiations, and regulators analyzed in the FANTOM5 project [60].

A list of transcription factor (TF) transcription start sites (TSSs) with enriched expression in Caco-2 cells was obtained from the FANTOM5 database (https://fantom.gsc.riken.jp/5/sstar/Browse_samples—data accessed on 15 January 2025) and compared to the list of TF-associated CAGE TSSs that were differentially methylated following IND10 and IND50 treatments. Expression values (in Tags per Feature, TF) were retrieved for all available samples (889 in total) across 23 selected promoters. The expression data were normalized using Z-score transformation. To simulate the transcriptional impact of differential methylation, three synthetic Caco-2 cell profiles were generated:

1. Maximum effect: minimum TF value for hypermethylated TSSs and maximum TF value for hypomethylated TSSs.
2. Medium effect: for hypermethylated TSSs, TF = TF_Caco-2—(median − minimum)/4; for hypomethylated TSSs, TF = TF_ Caco-2 + (maximum − median)/4.
3. Low effect: for hypermethylated TSSs, TF = TF_ Caco-2—(median − minimum)/8; for hypomethylated TSSs, TF = TF_ Caco-2 + (maximum − median)/8.

The original dataset, including the 889 samples and the three simulated profiles, was filtered to remove duplicate samples, resulting in a final dataset of 592 unique samples plus the three synthetic cell states. Hierarchical clustering analysis was then performed in R using Euclidean distance and complete linkage method.

### 3.7. Methylation Analysis of Repetitive Elements

Differentially methylated cytosines (DMCs) were annotated with respect to major classes of repetitive DNA elements using the *genomation* package in R. The analysis focused on short interspersed nuclear elements (SINEs), including Alu and MIR elements; long interspersed nuclear elements (LINE-1 and LINE-2); microsatellites; and simple repeats. The same package was applied to refine the annotation of DMCs located on chromosome 21, including DNA elements, simple repeats, long terminal repeats (LTRs), satellites, low-complexity regions, transfer RNAs (tRNAs), and ribosomal RNAs (rRNAs).

Statistical data regarding the distribution of DMCs among different repeat classes were retrieved from the UCSC Table Browser. The CpG content for each repetitive element family was calculated using the Biostrings package in R, which enabled analysis of multifasta files containing sequences grouped by repeat family.

## 4. Conclusions

This study provides a genome-wide view of DNA methylation changes induced by Indicaxanthin in Caco-2 intestinal epithelial cells. We observed significant alterations in DNA methylation at promoter regions, including genes involved in metabolism, inflammation, and transcriptional regulation. These modifications are consistent with a potential shift in the cellular program from a proliferative state toward a more quiescent or differentiated phenotype.

Notably, we detected consistent hypermethylation at ribosomal DNA loci, which may impair ribosome biogenesis and contribute to nucleolar stress and autophagy activation. We also found a preferential modulation of repetitive elements on chromosome 21, opening new perspectives for further research on the epigenetic sensitivity of this genomic region.

While the results support the idea that Indicaxanthin may influence cell identity through epigenetic remodeling, we acknowledge that a functional demonstration of phenotype conversion or transdifferentiation is not provided in this study. Further investigation will be necessary to clarify the biological consequences of these epigenetic changes.

Overall, our findings suggest that Indicaxanthin acts as an epigenetically active compound capable of reshaping regulatory regions of the genome, reinforcing its potential as a bioactive dietary molecule of therapeutic interest.

## Figures and Tables

**Table 1:** Analysis of the annotated DMRs.

	tot	% (tot)	Hyper	% (Hyper)	Hypo	% (Hyper)
Genecode.prom	1761	54	800	53	961	55
CGI	1706	52	775	52	931	53
CGI shores	652	20	324	22	328	19
CCRE	2512	77	1113	74	1399	80
DHS	1380	42	588	39	792	45
CTCF	235	7	96	6	139	8
H3K4me1	616	19	281	19	335	19
H3K4me3	1570	48	696	46	874	50
H3K9me3	42	1	22	1	20	1
H3K36me3	2	0	2	0	0	0
TSS.CAGE	612	19	283	19	329	19
CAGE.prom	2060	63	927	62	1133	65

**Table 2:** Differentially methylated gene types.

Type	%	Type	%	Type	%
protein_coding	61.6	misc_RNA	2.0	rRNA	1.0
lncRNA	29.3	TEC	1.4	snoRNA	0.3
miRNA	2.3	snRNA	1.3	other	0.5

**Table 3:** The most hypomethylated genes in their promoters (IND50 and IND10, diff.meth −100:−70).

Gene	diff.meth IND50	Type	Description	Tumor Function
*ACY3*	−70.8	coding	aminoacylase 3	
*C19orf73*	−82.9	coding	chromosome 19 open reading frame 73	
*CABIN1*	−74.6	lncRNA	calcineurin binding protein 1	Potential oncogene
*DIO3*	−81	coding	iodothyronine deiodinase 3	
*DSCAM*	−80	coding	DS cell adhesion molecule	
*FXYD7*	−78.1	coding	FXYD domain containing ion transport regulator 7	
*LINC01551*	−72.5	lncRNA	long intergenic non-protein coding RNA 1551	
*ONECUT1*	−96	coding	one cut homeobox 1	tumor suppressor
*PROK2*	−73.3	coding	prokineticin 2	
*RAP1GAP2*	−71.2	coding	RAP1 GTPase activating protein 2	tumor suppressor
*SHC2*	−80.2	coding	SHC adaptor protein 2	oncogene
*SHH*	−88.6	coding	sonic hedgehog signaling molecule	oncogene
*ZNF525*	−100	coding	zinc finger protein 525	

**Table 4:** The most hypermethylated genes in their promoters (IND50 and IND10, diff.meth +100:+80).

Gene	diff.meth IND50	Type	Description
*BARX2*	86.3	coding	BARX homeobox 2
*CD72*	83.3	coding	CD72 molecule
*CHFR*	91.6	coding	checkpoint with forkhead and ring finger domains
*CYP1B1-AS1*	92.7	lncRNA	CYP1B1 antisense RNA 1
*EFCAB6*	84.6	coding	EF-hand calcium binding domain 6
*MSX2*	95.2	coding	msh homeobox 2
*NAPRT*	88.8	coding	nicotinate phosphoribosyltransferase
*PTGIS*	100	coding	prostaglandin I2 synthase
*RHBDL1*	90.9	coding	rhomboid like 1
*RNU6-765P*	85.5	snRNA	RNA, U6 small nuclear 765, pseudogene
*STAT5A*	89.5	coding	signal transducer and activator of transcription 5A
*TIMP2*	85.3	coding	TIMP metallopeptidase inhibitor 2
*TMEM200C*	85.2	coding	transmembrane protein 200C
*UGDH*	81	coding	UDP-glucose 6-dehydrogenase
*ZKSCAN5*	93.9	coding	zinc finger with KRAB and SCAN domains 5

**Table 5:** Enrichment in transcription factor target genes.

Enrichment FDR	nGenes	Pathway Genes	Fold Enrichment	Pathways
9.4 × 10^−6^	12	27	6.5	*SOX10* target gene
1.2 × 10^−5^	61	440	2	*TFAP4* target gene
2.2 × 10^−8^	127	1041	1.8	*PAX5* target gene
7.9 × 10^−7^	127	1107	1.7	*AHR* target gene
3.1 × 10^−5^	108	970	1.6	*NFKB1* target gene
5.3 × 10^−9^	194	1757	1.6	*ARNT* target gene

**Table 6:** List of genes and promoters enriched in Caco-2 cells and with different methylation after IND treatments.

Gene	Entrez Gene	Diff Meth Prom	Meth.Diff IND50
**HYPER**			
*ASCL2*	430	all	62.1
*EPAS1*	2034	all	62.1
*FOXA3*	3171	all	44.8
*HOXA5*	3202	all	55.1
*HOXB3*	3213	p1+p2+p4@HOXB3	37
*HOXC4*	3221	p3@HOXC4	39.8
*IKZF4*	64375	p1+p3@IKZF4	42
*KDM2A*	22992	p1@KDM2A	41.1
*MSX2*	4488	all	95.2
*NR4A1*	3164	p3@NR4A1	67.7
*SALL4*	57167	all	35.5
*SOX13*	9580	all	27.6
*SP6*	80320	p1+p2@SP6	34
*TBX20*	57057	all	25.1
*TBX3*	6926	all	56.5
*TOE1*	114034	all	29.2
*ZKSCAN5*	23660	all	93.9
*ZNF93*	81931	all	58.9
**HYPO**			
*TBX1*	6899	p1+p5@TBX1	-32.3
*MYBL2*	4605	all	-39.9
*EGR3*	1960	all	-38.9
*ZNF331*	55422	p4+p7@ZNF331	-98
*THAP11*	57215	all	-29

**Table 7:** Annotation of DMC in repetitive elements.

	IND10 Hyper	IND10 Hypo	IND50 Hyper	IND50 Hypo
All DMC	99707	80526	92256	60594
All DMC in RE	52043	40970	50691	33452
Chr21 DMC	1918	939	3178	808
Chr21 DMC in RE	1018	509	1784	482

**Table 8:** Proportions of different classes of repetitive sequences in the genome and in chr21.

Track	CLASS	Family	Genome (%)	Chr21 (%)
RepeatMasker	LINE	L1	17.7	15.6
RepeatMasker	LINE	L2	3.5	2.3
RepeatMasker	SINE	Alu	10.6	8.8
RepeatMasker	SINE	MIR	2.8	1.6
RepeatMasker	Other	Other	17.4	23.3
RepeatMasker	All repeats	-	52	51.6
Simple Repeats	simpleRepeat	-	4.8	9.2
RepeatMasker	Simple_repeat	Simple_repeat	1.3	1.8

**Table 9:** Track names and datasets ID used for the annotations.

Track	Table or Experiment ID
Genes	AllGencodev39 and RefSeq All (ncbiRefSeq)
CpG Islands	cpgIslandExt
ENCODE cCRE	encodeCcreCombined (2024)
TSS CAGE	hg38_liftover+new_CAGE_peaks_phase1and2
Dnase HS (Caco2)	wgEncodeRegDnaseUwCaco2Peak
ChIP-seq CTCF (Caco2)	ENCFF990ZZT
ChIP-seq H3K36me3 (Caco2)	ENCFF479TQU
ChIP-seq H3K27me3 (Caco2)	ENCFF649WIT
ChIP-seq H3K4me3 (Caco2)	ENCFF642BGI
ChIP-seq H3K4me1 (Caco2)	ENCFF095KMQ
ChIP-seq H3K9me3 (Caco2)	ENCFF945SLD

## Data Availability

Data available upon request.
